# Esophageal Electrical Cardioversion of Atrial Fibrillation: When Esophagus Gives a Help to Cardiologists

**DOI:** 10.4061/2011/983937

**Published:** 2011-09-15

**Authors:** Luca Santini, Giovanni B. Forleo, Francesco Romeo

**Affiliations:** Division of Cardiology, Internal Medicine Department, University of Rome “Tor Vergata,” 00133 Roma, Italy

## Abstract

Atrial fibrillation is a common clinical disease especially in the elderly and in patients with organic heart disease. Electrical cardioversion is the first choice therapeutic approach for patients in which sinus rhythm could improve the quality of life and where the maintenance of sinus rhythm is considered likely. There are different techniques to perform an electrical cardioversion, each with specific indications, advantages, and limitations. The method most frequently used to restore sinus rhythm is external direct current cardioversion; however, this technique has some disadvantages, since it requires a high energy and usually general anesthesia. Esophageal cardioversion is an alternative method to obtain restoration of sinus rhythm, warranting acute and long-term results absolutely comparable with those obtained by the conventional transthoracic technique, especially in obese and COPD patients with high thoracic impedance for whom the standard technique may be less effective.

## 1. Introduction

Atrial fibrillation is a common clinical disease especially in the elderly (3–5% of the population over 60 years), and in patients with organic heart disease (70–80%) [1]. 

Electrical cardioversion (ECV) is the first-choice therapeutic approach for patients in which sinus rhythm could improve the quality of life and where the maintenance of sinus rhythm is considered likely. This technique compared with pharmacological cardioversion shows some important advantages: immediate effect, high success rate, and safety in hemodynamically unstable patients.

There are three main groups of patients in whom sinus rhythm is a benefit:

- patients with severe symptoms during the arrhythmia,
- patients with recent-onset atrial fibrillation in order to prevent electrical remodeling,
- patients with structural heart disease, such as hypertension and ventricular hypertrophy, which can achieve a significant hemodynamic improvement by restoring sinus rhythm.

There are several techniques to perform an electrical cardioversion, each with specific indications, advantages, and limitations. The method most often used to restore sinus rhythm is the external direct current cardioversion; however, this technique has some disadvantages, since it requires a high energy and usually general anesthesia.

## 2. Esophageal Electrical Cardioversion

This type of cardioversion may overcome some limitations of the standard external cardioversion. In some patients, the high thoracic impedance, due to emphysema or to a high body surface, changes the transmission of direct current shock through the thorax and represents a significant predictor of failure of external cardioversion [2]. Esophageal cardioversion provides several advantages such as the following:

- a lower energy requirement thanks to closeness of the esophagus with the left atrium which warrants a lower energy dispersion and a lower defibrillation impedance. When we give a direct current shock, using an external configuration, only around 20% of energy delivered reaches the heart, because most of the energy is dispersed in noncardiac tissues, especially in high-thoracic impedance patients [3];
- avoidance of general anaesthesia or deep sedation: as low energies are required, a mild sedation is sufficient to make the procedure well tolerated by most of the patients;
- safety in patients with pacemaker or ICD: there is a lower risk of damage and of increasing the pacing threshold, which is a phenomenon related to shock intensity, especially dangerous for pacemaker-dependent patients [4];
- availability of atrial pacing backup: the esophageal catheter may also be used to stimulate the atrium in case of a prolonged postshock sinus arrest, sinus bradycardia, or a pacemaker exit block.

**Figure 1:**
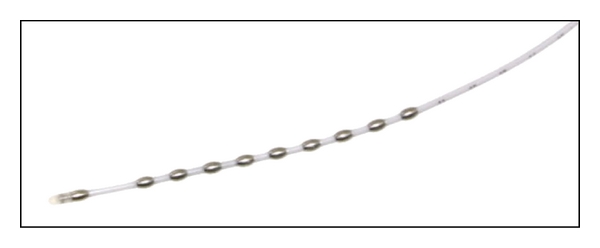
Decapolar catheter Esoflex, FIAB, Vicchio, FI, Italy.

**Figure 2:**
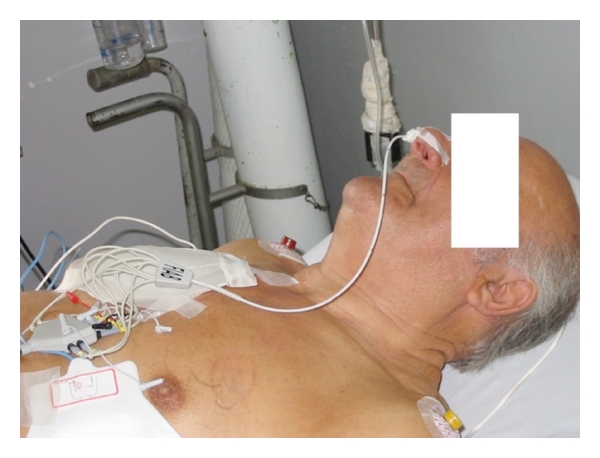
Esophageal-precordial configuration.

The technique used in the most recent studies about esophageal cardioversion is the esophageal-precordial cardioversion. In this configuration, energy is applied between the electrodes of an esophageal decapolar polyurethane catheter (5.7 cm^2^ total electrode surface, Esoflex, FIAB, Vicchio, FI, Italy) as cathode (Figure 1) and one or two precordial adhesive patch electrodes as anode. Such a configuration provides a greater electrode surface and embraces a larger area of atrial tissue (Figure 2). This configuration generates a uniform electric field during the shock which results in vectors with a low atrial defibrillation threshold.

Many studies, in more than 40 years of esophageal cardioversion, have proven safety of such technique, performing even histological examination of the esophageal mucosa in the animals underwent to esophageal shocks [5] or esophageal endoscopy in patients underwent to the esophageal-intracardiac cardioversion [6]. McKeown et al. [7] showed that no damage or dysphagia was seen in patients receiving shocks less than 100 joules.

Esophageal cardioversion is highly effective (95.3%). Furthermore, using 50 J or less, the 88.5% of the patients may be cardioverted [8]. 

The method is quite simple and very fast, and the only criterion used to assess the good position of the catheter can be the length of the catheter's part introduced into the esophagus (40–45 cm from the nostril), without any need of radioscopic control neither of recording the esophageal ECG. The sedation may be obtained by different drugs, the most used is midazolam, which is effective at low dosage, safe, and handy. Finally, this technique showed to be well tolerated by patients and could be easily performed in an outpatient regimen. 

Recently, our group has compared the external electrical cardioversion and the esophageal one, both under a conscious sedation by midazolam. The conclusions were that the outpatient cardioversion of AF may be performed safely and effectively by either a transthoracic or a transesophageal approach. The rate of early recurrence of AF before the end of sedation did not show any significant difference between the two groups, and a second ECV was effective in all the patients [9]. As transesophageal ECV shows no clear advantage, transthoracic cardioversion should remain the approach of first choice, due to the economical issues and to the lowest complexity. 

Nevertheless, esophageal cardioversion may still play an important role in selected patients such as obese or COPD patients with high thoracic impedance. 

A particular further advantage in the use of the transesophageal approach for the ECV could be the possibility to use a probe which combines the echocardiographic imaging capabilities of a probe in the esophagus with the cardioverting abilities. Two publications have outlined this approach [10, 11]. In these two papers, authors showed as a custom-made probe, for combined TEE plus TEC, offered an effective early cardioversion with low energy levels after exclusion of a clot. The procedure was well tolerated, and even hemodynamics could be monitored during and immediately after cardioversion. These two papers show how, with such an approach, it could be possible to perform in a unique step two procedures that at present are necessarily performed through two separate steps: first, the exclusion, by a TEE probe, of the presence of a left atrial thrombus and then the execution, by the same probe introduced into the oesophagus of the electrical cardioversion. Such an approach, of course, would have the clear advantage to be time saving and cost effective.

## 3. Conclusions

There are different techniques to perform an electrical cardioversion, each with specific indications, advantages, and limitations. The method most frequently used to restore sinus rhythm is the external cardioversion, which showed to be a safe, effective, and well-tolerated technique even avoiding general anaesthesia or deep sedation, especially now that biphasic waveform defibrillators are widely available [9, 12, 13]. Nevertheless, the esophageal cardioversion may still play an important role in obese and COPD patients with high thoracic impedance for which the external one may be less effective. A very promising application for esophageal electrical cardioversion could arise from the possibility to use a probe assembled for simultaneous transesophageal echocardiography and transesophageal cardioversion [10, 11]. The use of such a combined probe may be the technique of choice for patients who require both cardioversion and transesophageal echocardiography.

Therefore, esophagus comes again to help cardiologists as it does, on a routine basis, since more than 20 years through the transesophageal echocardiography [14, 15] or the transesophageal electrophysiological study [16], offering them an alternative, safe, and very effective technique to perform electrical cardioversion and restore sinus rhythm.
